# Progression of Vertebral Compression Fractures After Previous Vertebral Augmentation: Technical Reasons for Recurrent Fractures in a Previously Treated Vertebra

**DOI:** 10.7759/cureus.1776

**Published:** 2017-10-16

**Authors:** Robert E Jacobson, Ovidiu Palea, Michelle Granville

**Affiliations:** 1 Miami Neurosurgical Center, University of Miami Hospital; 2 Anesthesiology and Pain Management, Provita Hospital

**Keywords:** osteoporotic vertebral compression fractures, kyphoplasty, vertebral augmentation, recurrent vertebral compression fractures, vertebral fracture clefts, aseptic necrosis of the vertebrae, repeat vertebral fracture, progressive vertebral fracture, post-vertebroplasty fractures

## Abstract

It is well recognized that patients can develop additional vertebral compression fractures (VCF) in an adjacent vertebra or at another vertebral level after successful vertebral augmentation. Factors such as the patient's bone mineral density, post procedure activity, and chronic corticosteroid use contribute to an increased risk of re-fracture or development of new fractures in the first three months after the initial procedure. However, there is a very small subgroup of patients that have unchanged or worse pain after the vertebral augmentation that may indicate continued progression of the treated compression fracture or a recurrent fracture at the previously treated level. This review examines the clinical findings, radiologic signs, and intraprocedural technical failures that may occur during the initial vertebral augmentation that can lead to a progressive fracture in a previously treated vertebra. Causes of failure of the initial vertebral augmentation procedure include inadequate or incomplete filling of the fracture site, the cement missing the actual fracture allowing continued osteoporotic compression, and persistent or worsened intravertebral fluid-filled clefts. The existence of an unfilled intravertebral fluid cleft on preoperative diagnostic studies is the most important indicator of risk for progression as is the later development of fluid at the bone cement interface.

## Introduction and background

Between 20% to 35% of patients with osteoporotic vertebral compression fractures later develop other vertebral fractures after treatment with vertebral augmentation, usually related to another traumatic event [1]. This repeat fracture risk continues to increase over time with or without intervening vertebroplasty or kyphoplasty, especially if patients are not on medical treatment for their underlying osteoporosis [1-2]. New pain after initial improvement can be an indication of either a new fracture at another spinal level or a fracture adjacent to the level of the previous augmentation [2]. Studies have documented that the larger volumes of cement used to maintain sagittal alignment after balloon kyphoplasty may actually increase the risk of new adjacent level fractures due to increased rigidity of the treated vertebral body adjacent to an already osteoporotic vertebra [3-4]. It has also been noted that intra-discal leakage of cement can also increase the risk of adjacent level fracture for a similar reason [5]. These later developing adjacent level fractures are often labeled as 'recurrent' fractures but are quite different than fractures that progress in previously treated vertebrae [4-5]. The few reports focused on the progression of treated fractures observed that many cases do not have a new traumatic event and frequently have an additional finding of a fluid-filled cleft adjacent to the original osteoporotic fracture on computerized tomography (CT) and magnetic resonance imaging (MRI) [6-7]. These clefts are also described as areas of aseptic necrosis and intravertebral instability showing clear enlargement on dynamic films with lumbar extension [7]. Several large studies of vertebral augmentation and vertebral compression fractures (VCF), while noting the small incidence of progressive fractures at a treated level, have not examined the possible technical reasons for the progression of the fracture and specific causes of failure after injection of cement that should stabilize the fracture [8-10]. This review will focus on examples of progressive fractures where the radiologic findings after the initial vertebral augmentation indicated possible technical reasons that could explain why the initial vertebral augmentation failed, leading to the progression of the VCF. Understanding the reasons for failure additionally helps to plan the best approach and location within the fractured vertebrae for a repeat procedure. Repeat vertebral augmentation targeting the parts of the vertebrae not reached by the cement in the original vertebral augmentation is a reasonable solution to prevent further vertebral collapse and relieve pain.

## Review

Studies demonstrate between 75% to 90% excellent to good pain relief within seven to 10 days after vertebral augmentation whether performed with a balloon or injecting cement with or without cavity formation [1-3]. Long-term results can be affected by the severity of the underlying osteoporosis and chance of repeat fractures [1-3]. Review of the literature regarding repeat fractures at the same level can be confusing since many articles that review and discuss repeat fractures actually are only discussing new fractures at an adjacent or different level and not at the same level [2, 4, 6, 9]. In a longitudinal 12-month follow-up study after vertebral augmentation, 12% of patients developed adjacent level fractures with a mean time from treatment of 68 days, 23% developed fractures remote from the treated vertebrae with a mean of 100 days, and 19% had further collapse of the treated vertebrae in an average mean of 78 days [11].  Persistent or worsening pain after vertebral augmentation, especially without a new fall or trauma, is characteristic of osteoporotic fracture progression [1-2, 9-10].

Radiologic findings seen with recurrent fractures using radiographs, computerized tomography (CT), and magnetic resonance imaging (MRI) show a progressive decrease of vertebral height, increased angulation associated with increased vertebral edema in the treated vertebrae, and evidence of the development or persistence of a fluid-filled vertebral cleft [7, 9-12]. However, it is important to note that several long-term studies post-vertebral augmentation show 10% to 15% gradual height loss in the treated vertebrae in up to 30% of the patients with follow-up between 12 and 24 months. This is secondary to the underlying osteoporosis within the other parts of the vertebrae not treated with cement [1-2, 5]. In patients with recurrent fractures in the same vertebrae, the loss of vertebral height is much more acute and patients are usually symptomatic within the first 60 to 90 days post-procedure [5, 11]. Paradoxically, it has been observed that when there is a marked increase in the anterior vertebral height after the procedure there is actually a higher risk of future collapse, suggesting that it may not be advantageous to try and get maximum restoration of vertebral height, especially with kyphoplasty [6, 9]. Multisection CT often can detect fracture lines into the endplates and posterior vertebrae wall that are related to a higher incidence of both leakages into the disc space and intervertebral canal and the epidural space. Studies have shown that the risk of persistent pain and potential re-fracture can be extrapolated from this information [13]. Leakage of cement is consistently underestimated based on intraoperative radiographs. Follow-up CT scans after augmentation in large patient groups have shown up to a 45% incidence of asymptomatic leaks, both into the epidural space and the adjacent disc space [14]. The majority of these leaks are minor and do not cause symptoms, but an excessive volume of cement leakage, especially into the adjacent disc space, is critical to an increased risk of adjacent level fractures [6, 9, 12, 14].

Persistent pain after a vertebral augmentation can be caused by different spinal problems, including another osteoporotic compression fracture at a new or adjacent level, surgical infection, concurrent lumbar stenosis, and rarely, a recurrent fracture at the previously treated level [11, 15]. There are case reports of the development of an adjacent level fracture as early as the first month after kyphoplasty [3, 6]. Multiple fractures and development of sequential cascading osteoporotic fractures are well recognized due to severe underlying osteoporosis. Multiple fractures are detected at the time of initial evaluation in 10% to 15% of the reported series, and the chance of further fractures can be as high as 35% [1-3, 12]. However, the reported incidence of continued progression of a VCF after vertebral augmentation is relatively small, ranging from 0.56% to 2% [8-11]. Patients still complaining of significant spinal pain after undergoing a vertebral augmentation must be reevaluated with plain radiographs, CT, and MRI scans. Clinically, most patients that have fracture progression do not have a new injury which distinguishes them from repeat fractures at new levels [9-12]. Previous reports of repeat fractures at the same treated level have focused on the incidence of repeat fractures and the significance of vertebral clefts [8-12]. None of the reports focused on identifying possible technical reasons why the first vertebral augmentation failed to stop or stabilize the VCF.

A recurrent fracture at a level previously treated with kyphoplasty or vertebroplasty is very rare, varying from less than 1% to 2% of cases in large series [4, 8-9]. In one of the largest studies reported with a two-year follow-up study of 1,800 patients, only 10 or 0.56%, developed a recurrent same level fracture after vertebroplasty [11]. Even though a recurrent fracture at the same vertebrae occurs in a very small percentage of patients, it accounts for many of the patients that have very poor results [2, 9]. Technical issues at the time of the initial vertebral augmentation may contribute to the failure of the initial vertebroplasty leading to continued progression of the VCF and further vertebral collapse.

Specific examples of cases seen after the failure of the initial vertebral augmentation and kyphoplasty are reviewed where there was rapid fracture progression. All the examples had previous multiple fractures, very poor bone mineral density despite being on medical treatment, no new precipitating fall or injury after the vertebral augmentation, and on a review of follow-up radiologic studies, there were identifiable technical reasons for the failure of the initial procedure.

Progression of a fracture after only partial filling of the fractured vertebral endplate 

**Figure 1:**
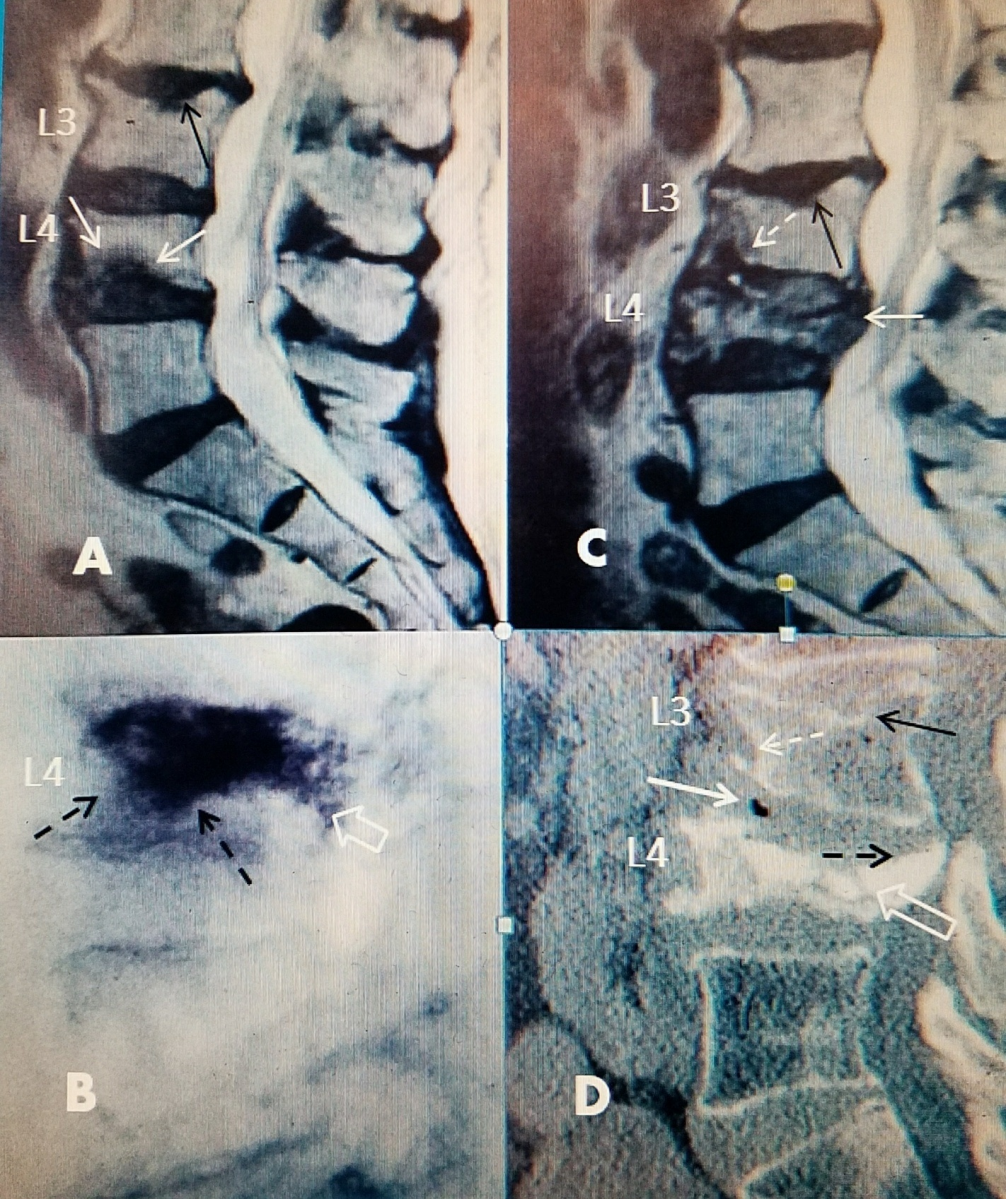
Progression of L4 inferior endplate fracture after vertebral augmentation A: Sagittal T2 magnetic resonance image (MRI) showing inferior anterior endplate edema at L4 (white arrows) and an old minimal superior endplate fracture at L3 (black arrow). B: Intra-operative films after bilateral vertebral augmentation showing diffuse spread of bone cement (empty white arrow). There is spread of bone cement under the entire superior endplate into inferior anterior vertebral body toward the fracture site (dotted black arrows). C: Four weeks later, there is progressive collapse of L4 now associated with protrusion of posterior wall of the vertebral body into the spinal canal (solid white arrow). At the same time, a new edematous change has developed in the adjacent vertebrae at the inferior anterior part of L3 (dotted white arrow). This was not present on the initial MRI scan. There is also the beginning of very slight kyphotic angulation at L3 over L4 that was not seen on the initial scan. D: Computerized tomography (CT) six weeks after the initial procedure showing further progressive collapse of L4 (hollow white arrow). There is also a new vacuum change in the anterior part of the L3-4 disc space (solid white arrow). There is developing collapse anterior inferior at the L3 vertebrae (dotted white arrow). The L3 superior endplate fracture is unchanged (solid black arrow).

In Case 1, a 78-year-old female with severe osteoporosis and a bone mineral density (BMD) of -2.4 while on bisphosphonates developed a painful fracture of the inferior endplate of L4. She had multiple previous fractures at the superior endplate of L3, T9, and T11. After three months of conservative treatment with a lumbar brace because of increasing pain, she underwent unilateral balloon kyphoplasty with polymethylmethacrylate (PMMA) at the L4 vertebra. Initial postoperative radiographs showed a partial fill of L4 with cement not completely filling the inferior endplate of the vertebrae where the fracture and edema were primarily located. The patient initially had about 50% decrease in pain but within two weeks spontaneously developed increased pain in the same location. An MRI scan performed four weeks after the vertebral augmentation showed the progressive collapse of the L4 vertebra with new posterior protrusion of the vertebrae into the spinal canal associated with edema of the anterior part of L3. The new change at L3 was indicative of the early development of an adjacent level fracture. She refused further procedures and continued wearing a thoracolumbar support. Follow-up CT scan showed a continued collapse at L4 with the progression of the fracture, the development of a vacuum cleft in the disc space at L3-4, and further adjacent level fracture of the anterior part of L3 (Figure 1).

This example highlights that progression of the collapse after vertebral augmentation can occur very quickly and lead to a secondary fracture of the adjacent vertebral level. Often, there can be 'occult' progression and the subsequent development of edema on MRI scan. There does not necessarily have to be a progressive change in vertebral height or clear evidence of radiologic collapse [16-17]. However, a gradual height decrease, combined with persistent or worsening pain, should raise the suspicion of fracture progression. However, follow-up studies over 12 to 24 months have shown up to 20% of patients will continue to have further height loss without pain [2, 5, 18]. Vertebral height correction with balloon kyphoplasty can also be lost within the first 90 days in up to 25% of cases [2, 18]. There have been studies examining if the location, amount, and type of cement injected affected the outcome of vertebral augmentation. The distribution of cement is important since failure to obtain an adequate spread of the cement into the fractured area and specifically near the fractured endplates may lead to further collapse [19]. Even hemivertebral filling does not affect the risk of recurrent fractures at adjacent levels [20]. Biomechanical studies show the cement adds general stiffness to the vertebral body and improves the in vivo resistance to experimental vertical compression [21]. There are two types of cement used for vertebral augmentation. Polymethylmethacrylate (PMMA) cement is an inert hydrophobic polymer and Cortoss® (Stryker®, Malvern, PA), which is a bioactive calcium phosphate micro-glass cement that is more bone binding and hydrophilic. The hydrophilic quality has better flow characteristics than traditional PMMA [22-23]. Biomechanically, Cortoss is stronger than PMMA and more similar in strength to cortical bone. The material is osteoconductive so over time there will be better osteointegration. We chose to use Cortoss for the following repeat vertebral augmentation procedures because of the more hydrophilic nature of Cortoss and its less viscous flow characteristics compared to PMMA [22-23]. This is important in repeat procedures for recurrent fractures where there is incomplete fill around the PMMA since this enables the surgeon to get a better fill of cement into the trabecula in both the residual fracture and, more importantly, into the bone supporting the endplates of the vertebrae.

Cement inadequately filling the fracture site and leakage into the adjacent disc space

**Figure 2:**
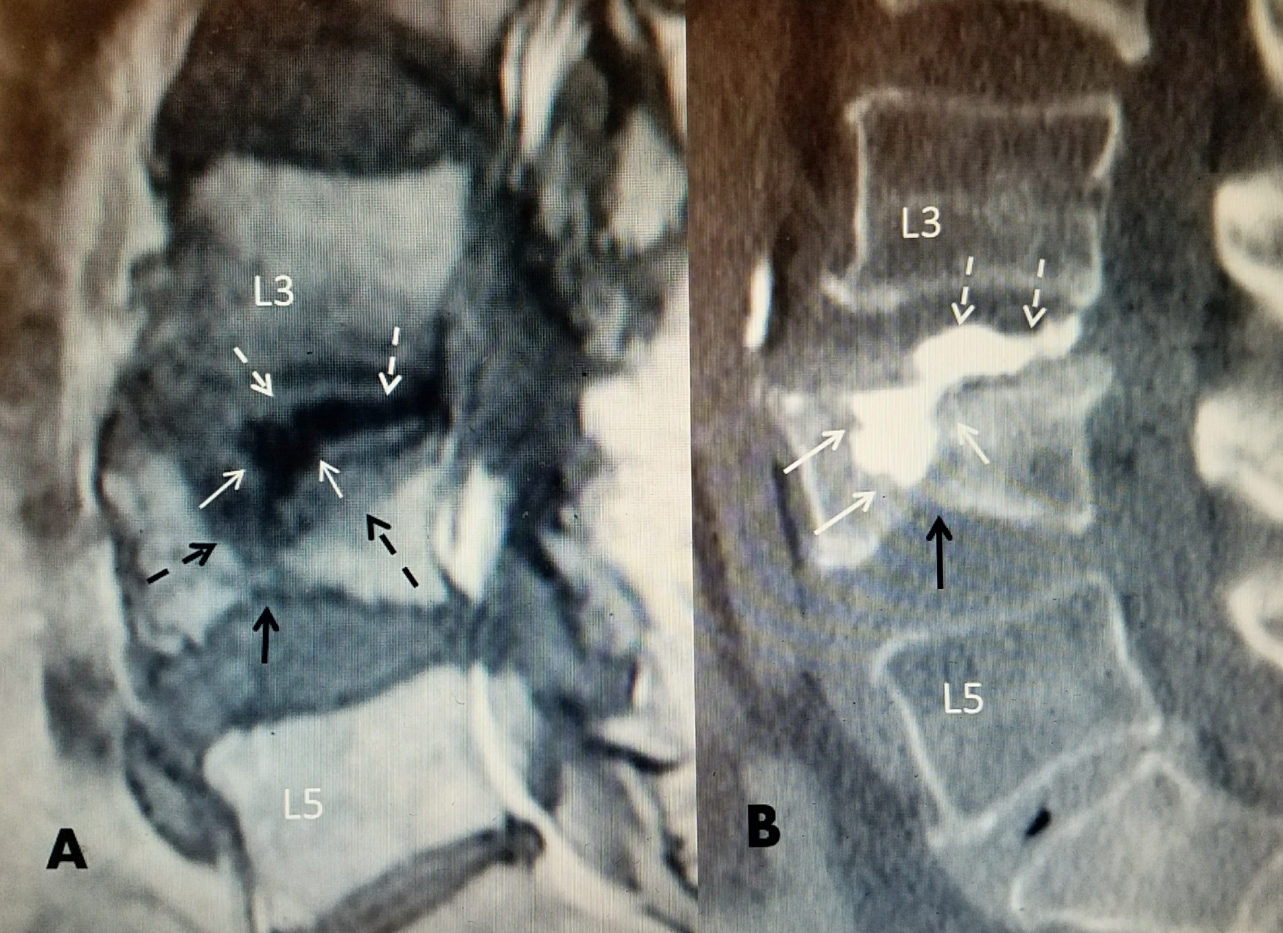
L4 wedge superior endplate fracture where cement inadequately filled the fracture A: T1 sagittal MRI post-L4 vertebral augmentation with polymethylmethacrylate (PMMA). There is only a partial fill of the large mid-vertebrae edematous fracture (dotted black arrows) with significant cement leakage into the middle and posterior L3-4 disc space (dashed white arrows). There is also an inferior endplate fracture (solid black arrow) creating biconcave vertebrae. B: Sagittal CT scan post repeat vertebroplasty. An extrapedicular approach was used to access the fracture site. Post repeat vertebral augmentation with Cortoss® at L4 showing an almost complete fill of the large midline fracture (solid white arrows). The small inferior endplate fracture is unchanged (solid black arrow). The previous leak of cement into the L3-4 disc space is unchanged (dashed white arrows).

In Case 2, a 67-year-old female on chronic corticosteroids for asthma had two previous lower thoracic-lumbar fractures treated with kyphoplasty at T11 and L1. She was on long-term bisphosphonates for severe osteoporosis with a BMD of -2.9. She developed a new L4 fracture after a fall. CT of the fracture showed a large central concave deformity in the middle of L4. She underwent kyphoplasty with PMMA but the cement only minimally filled the large midbody fracture. Her pain never improved, and over the next six weeks, the pain actually worsened. Repeat films showed there was minimal cement filling the middle and inferior part of the original fracture and the cement had also leaked into the superior adjacent disc space. The patient underwent a second vertebroplasty using Cortoss combined with curetting of the fracture site to allow better distribution and flow of the cement into the fracture. The cannulas used for injecting the cement were purposely angled toward the more inferior part of the fracture away from the disc space. The fracture was able to be filled with cement without any increase in the previous intra-discal extravasation of cement (Figure 2).

Within one day after the second vertebral augmentation, the patient was active and ambulating with minimal pain. This example demonstrates that often there is not complete control of where the injected cement is distributed. In a high percentage of cases, sufficient cement can be distributed bilaterally through a unipedicular approach with good results. Biomechanical studies show unilateral vertebroplasty creates sufficient vertebral stiffness to prevent further vertebral compression as long as it fills the actual fracture site [18]. In this example, there was also cement leakage into the adjacent disc space. Cement leaking into the disc space has been related to increased incidence of adjacent level fractures. Studies have found that both intra-discal and epidural leakage of small amounts of cement is much more common on follow-up post-procedure CT scans than routinely detected during intra-operative fluoroscopy [3, 5, 11]. If the cement is observed going into the disc space, this can lead to inadequate filling of the fracture, possibly leading to continued vertebral collapse. Technically, if the cement is extravasating into the disc space or if the injection is stopped and the cement is allowed to solidify, it is possible to redirect the cannula through the opposite pedicle or place a second cannula in a different position, even through an extra-pedicular technique to continue to fill the specific area within the vertebral fracture. In this case, during the second procedure, the vertebroplasty cannulas were directed toward the middle part of the vertebrae and, using curettes, space was developed around the previous PMMA to allow the Cortoss to fill the fracture.

Cement or balloon missing the fracture

**Figure 3:**
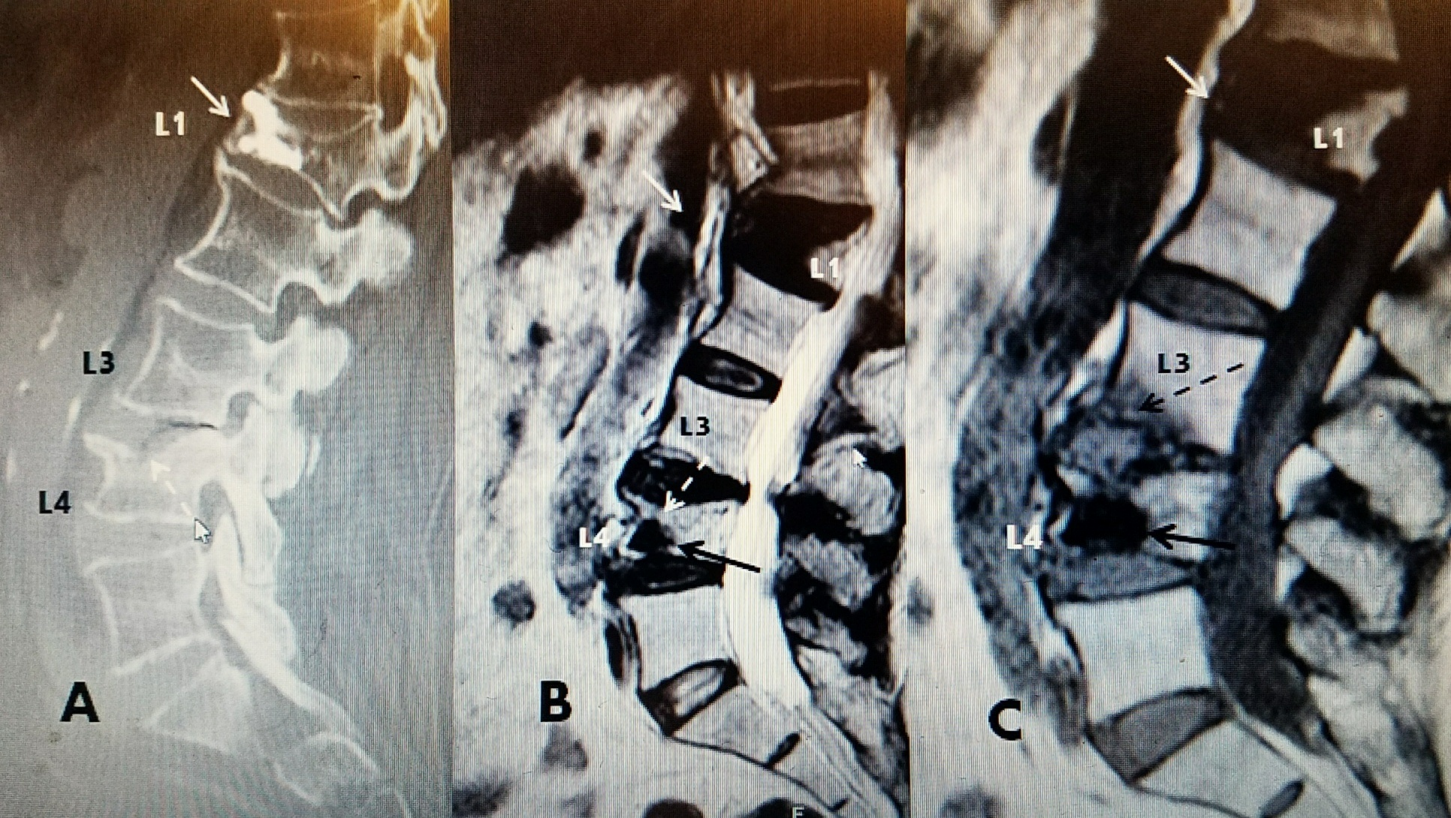
85-year-old male with an L4 compression fracture of superior endplate and kyphoplasty inferior to the fracture A: Computerized tomography scan (CT) showing L4 with a 20% anterior-superior endplate fracture (dotted back arrow). There was a previous vertebroplasty anteriorly at L1 (solid white arrow). B; Magnetic resonance image (MRI) T2 image post-kyphoplasty with polymethylmethacrylate (PMMA) showing cement anteriorly and inferiorly at L4 (solid black arrow), but below the fracture of the superior endplate (dotted white arrow). C: Sagittal MRI T1 one month after B showing new edematous change in the adjacent vertebrae in the anterior inferior part of L3 (dotted black arrow). The cement from the kyphoplasty is below the L4 superior endplate fracture (black arrow). The old L1 kyphoplasty cement is unchanged (solid white arrow).

Case 3 is an 85-year-old male who fell and developed a new inferior L4 endplate fracture. He lived on his own in an assisted living facility. He had a previous L1 fracture treated with a kyphoplasty. He had a bone mineral density of -2.6 and was not on any medical treatment for osteoporosis. A unilateral balloon vertebral augmentation with PMMA was performed but the balloon passed inferior to the superior endplate fracture. The cement never reached or filled the superior endplate fracture. The patient’s pain persisted and worsened over three weeks so that he became bedbound. Serial MRI scans showed progressive vertebral edema and additionally, the development of edema in the anterior inferior part of L3 adjacent to the L4 body (Figure 3).

**Figure 4:**
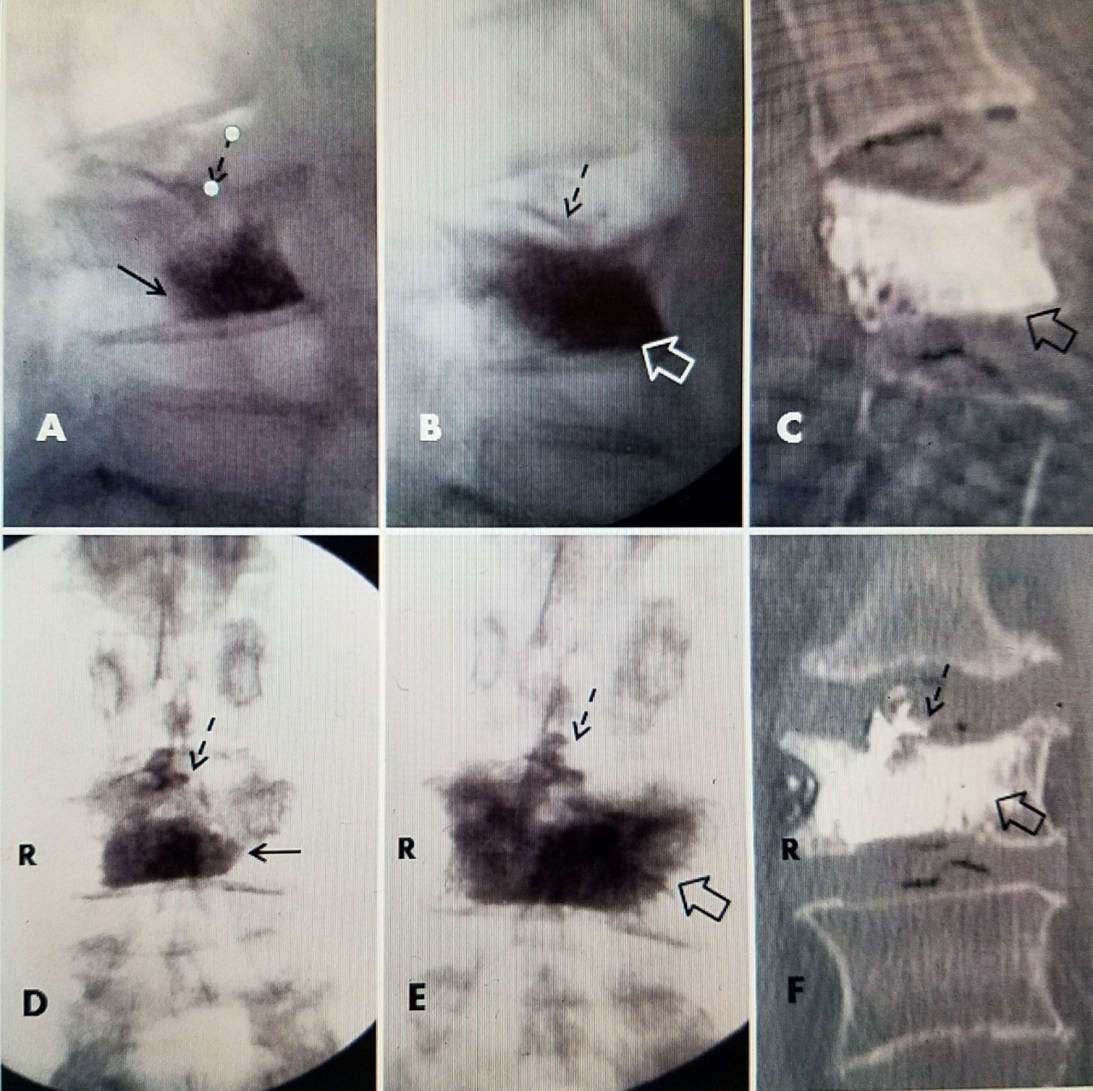
L4 kyphoplasty inferior and left and after bilateral vertebroplasty A: Initial intra-operative films of vertebral augmentation with balloon kyphoplasty using polymethylmethacrylate (PMMA): Lateral fluoroscopic image showing cement is only in the anterior and inferior body of L4 (solid black arrow) but definitely below and not in contact with the fracture of the superior endplate where a small amount of cement has extravasated (dotted black arrow) B: Lateral image after second vertebral augmentation using Cortoss® showing diffuse fill of the entire vertebrae, including the area adjacent to the fracture at the superior endplate (hollow white arrow). The intra-discal extravasation is unchanged from the first procedure (dotted black arrow) C: Sagittal computerized tomography (CT) after repeat vertebroplasty showing complete and diffuse fill of the vertebral body, including under the entire superior endplate and posterior wall of L4 (hollow black arrow). D: Anterior-posterior (AP) film after initial kyphoplasty showing only partial fill in the right inferior part of the vertebrae. There is minimal spread of cement below the fractured superior endplate (solid black arrow). There is intradiscal extravasation into the disc space at L3-4 (dotted black arrow). E: AP image after second procedure showing diffuse bilateral Cortoss® cement filling both sides of the vertebrae. There is better fill superiorly across the area of superior endplate fracture and inferiorly on the left (open black arrow). There is no change in the intradiscal extravasation at L3-4 from the first procedure (dotted black arrow). F: AP image of CT scan showing diffuse cement fill of L4 vertebrae (hollow black arrow). The intradiscal extravasation of cement at L3-4 is unchanged (dotted black arrow) from initial extravasation after the kyphoplasty as can be seen comparing images D, E, and F.

In this example, the technical reason for failure was that the original kyphoplasty went inferior to the L4 superior endplate fracture. As a result of the balloon placement, the cement was only injected anterior and on the left side, which was below the fracture. This led to a progression of the fracture and, secondarily, the development of edema in the anterior part of L3 adjacent to the fracture at L4. Within five weeks of the first procedure, bilateral vertebral augmentation was performed with Cortoss which filled the entire previously treated vertebra. The patient had rapid pain relief. This highlights that the surgeon needs to make sure the injected cement gets to the area of the fracture, the involved endplate, and the edematous part of the vertebra. Studies have shown that low volumes (up to three cc of cement) are sufficient to stabilize the compression if well distributed, especially along the fractured endplates [8, 17, 19-20]. In repeat cases, both the superior and inferior endplates should be treated to provide complete support to progressively collapsing vertebrae (Figure 4).

Inadequately filling a known vertebral cleft or development of cavitation around cement

**Figure 5:**
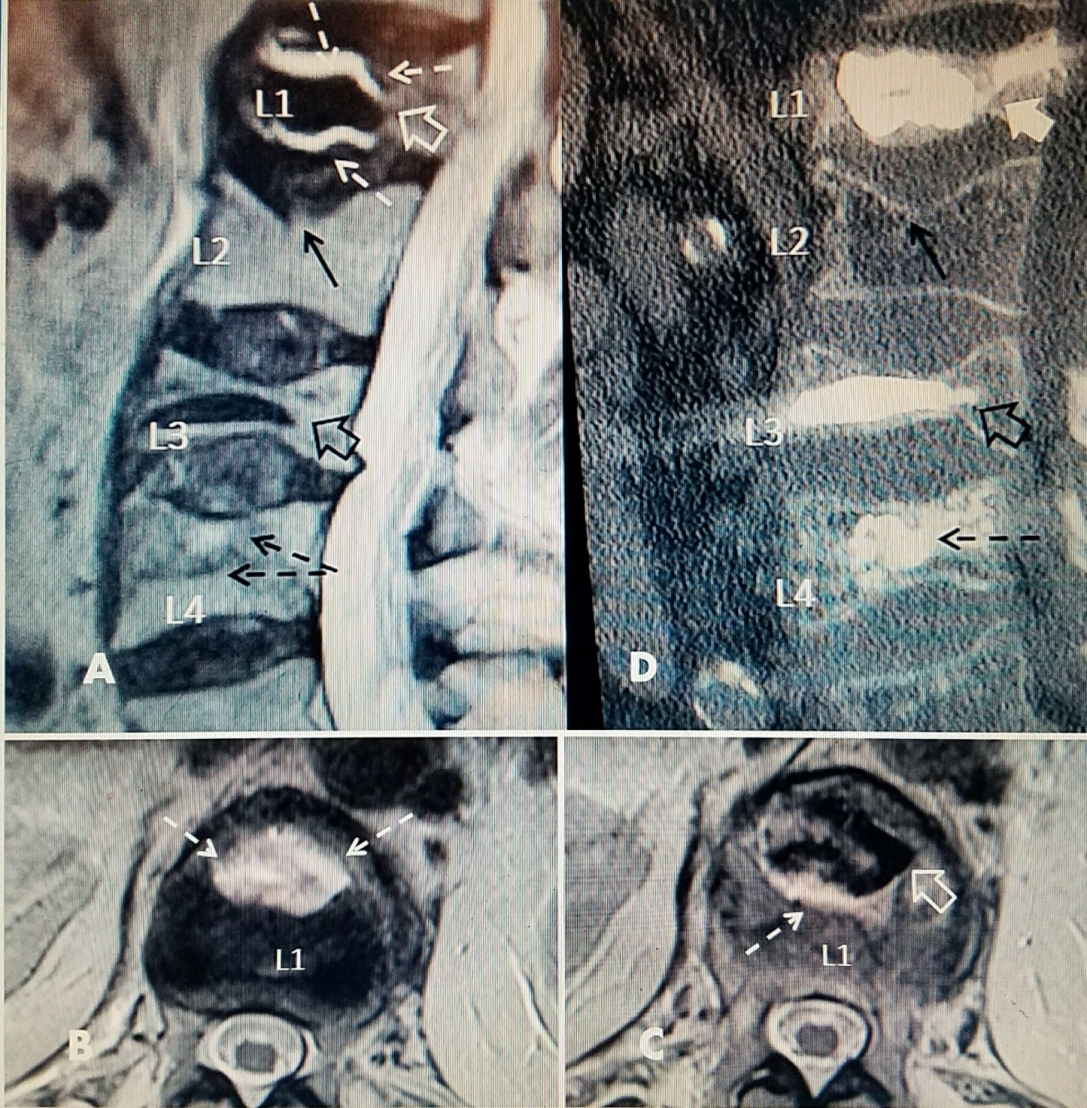
Repeat vertebral augmentation to fill post-kyphoplasty cleft A: Magnetic resonance imaging (MRI) post-kyphoplasty with polymethylmethacrylate (PMMA) at L1 showing fluid-filled vertebral cleft (dashed white arrows) surrounding the kyphoplasty cement (hollow white arrow). There is also a superior endplate fracture at L2 but no surrounding edema, indicating it is chronic (solid black arrow). Previous PMMA kyphoplasty cement is seen at L3 (hollow black arrow) and vertebroplasty cement at L4 (dashed black arrow). B+C: Axial T2 MRI images of L1 vertebrae after initial kyphoplasty showing the vertebral cleft and hyperintense fluid signal (dotted white arrows). The margin of PMMA cement (black) is seen in C (hollow white arrow). D: Sagittal computerized tomography (CT) after additional Cortoss® bone cement was percutaneously injected into the fluid cleft leading to a complete fill of the cavity at L1 so that the additional cement is incorporating the original kyphoplasty cement (full thick white arrow). The unchanged superior endplate fracture of L2 can be seen (solid black arrow). The previous kyphoplasty cement at the collapsed L3 is seen (hollow black arrow) and the scattered vertebroplasty cement is seen at L4 (dashed black arrow).

Case 4 involved an active 85-year-old male who had multiple previous kyphoplasties at T9, L3, and L4 with good pain relief. Ten months later, he had another fall and underwent an L1 kyphoplasty with PMMA. However, after the kyphoplasty at L1, he had continually worsening pain over three months. MRI scans revealed fluid and marked edema around the cement at L1. Review of the original pre-kyphoplasty MRI revealed a small cleft with a 30% wedge collapse of L1 and minimal fluid on MRI scan. The fluid-filled vertebral cleft or vertebral cleft worsened after the initial vertebral augmentation. PMMA kyphoplasty cement is a consistent finding associated with failure after an initial procedure. The fluid-filled cleft interferes with the interface between the cement and the fractured vertebrae. When performing a second procedure, the repeat procedure should be a vertebral augmentation using Cortoss rather than a kyphoplasty using PMMA. In this case, a curette was used to dissect around the previous kyphoplasty with PMMA and then Cortoss was injected bilaterally, which surrounded and stabilized the PMMA within the fluid-filled cavity in the L1 vertebral body. After this second vertebral augmentation, there was gradual pain relief over five days (Figure 5).

In several large studies, intra-vertebral clefts were identified in between 90% to 100% of cases of recurrent fractures in a previously treated level. In a large series of 1,800 cases, 10 had recurrent same level fractures and nine of the 10 had intra-vertebral fracture clefts [9]. Analysis of these cases found that excessive restoration of anterior height and intra-operative extension to create fracture alignment and provide a degree of kyphotic correction all were negative factors in the cases that developed a recurrent fracture. None of these 10 cases had a new injury but developed increased pain spontaneously; all were at the thoracic-lumbar junction, like this case example, and 90% had intervertebral clefts or fluid on CT and MRI scan [9, 24-26]. In a smaller study of 104 patients, six were found to have recurrent fractures at a previously treated level. All six cases also had intra-vertebral fluid on follow-up MRI scans [8]. Biopsy and aspiration of the clefts showed necrotic fluid similar to what is seen with aseptic necrosis [22, 24, 27]. Open revision surgery after failed vertebroplasty or kyphoplasty found minimal fibrotic reaction often associated with loose cement and fluid clefts. It was noted that a solid pattern of cement rather than a more diffuse trabecular pattern on postoperative films were seen in revision cases [28]. Biomechanical studies found that a more trabecular spread of cement distributes the load and stiffness throughout a wider area of the fractured vertebrae [22-23]. The importance of filling the cleft is the key step in preventing further collapse. The hydrophilic property of Cortoss enables it to be effective in filling the actual cleft either with previous PMMA, as was shown in a case with cavitation and failure around of a lumbar interbody graft, where the Cortoss filled the area around the graft and the adjoining cavity in the endplates from an interbody fusion [28]. Dynamic radiographs during surgery have shown that these intra-vertebral clefts enlarge when the patient's spine is placed in lordosis [23-26, 29]. Several authors stated that actual change in cyst size with movement indicates fracture instability and motion within the vertebrae [7, 9, 30-31]. In a symptomatic patient after an initial vertebral augmentation, if there is a clear fluid-filled cleft at the time of the initial procedure that did not completely fill with cement or the cleft develops or enlarges afterwards, then the cleft itself needs to be the target of the second procedure, along with the fractured endplate [25, 29, 31].  

Follow-up radiographs have noted between 10% to 15% asymptomatic settling of the vertebral body over the first 12 months after both vertebroplasty and kyphoplasty [8-9, 29, 31]. This is different than patients that develop progressive vertebral collapse combined with increasing or recurrent pain and have persistent or spreading intra-vertebral edema or intravertebral fluid clefts on MRI scan at follow-up. The overall incidence of the development of a symptomatic progressive or recurrent fracture at a previously treated vertebral fracture is around 1% to 2%. However, when recognized, it is a correctable cause of post-vertebroplasty back pain in progressively collapsing vertebrae. This article highlights the specific radiologic findings that may indicate a recurrent fracture, as well as possible technical reasons for a fracture to recur. If the original procedure may not have completely addressed the fracture site and the patient has worsening pain, redoing the original procedure is best based on where new CT and MRI studies show persistent fractures or fluid. The surgeon can also decide the best type of cement to use for these recurrent fractures. Previous articles have focused on incidence rather than the actual technical reasons that may be identified for failure. The technical reasons for initial vertebroplasty failure can affect the approach for a repeat procedure. In the three cases that underwent repeat vertebral augmentation, there was very good pain relief. In the one case that refused a repeat procedure, the vertebrae continued to collapse with persistent pain and eventual kyphotic deformity. In our small group and the reports in the literature, repeat vertebroplasty at the same level is safe and very effective.

## Conclusions

Persistent or worsening pain after vertebral augmentation or increasing pain after initial improvement can be an indication of possible fracture progression at the previously treated vertebra. Although new fractures after vertebral augmentation are usually at a level adjacent to a previous vertebral augmentation or a different level, there are distinct indications that the treated level may have re-fractured. Clinically, patients with fracture progression at the same level have increasing pain without any new trauma. Repeat studies with MRI and fine cut CT scans can demonstrate missed or inadequately filled fractures, inadequate filling of the fractured endplate, increasing vertebral edema, and especially the finding of fluid-filled intravertebral fracture clefts. It is important to recognize that a patient can have progression of the originally treated fracture and, in addition, may develop a fracture of the adjacent vertebral level at the same time. By analyzing the reasons for the failure of the original vertebral augmentation, it is possible to better plan the surgical approach for the repeat procedure. This report shows how these areas can be identified and then specifically targeted during a repeat procedure. Knowing where the additional cement needs to be placed and possibly using more hydrophilic cement, such as Cortoss, which provides better cement distribution in the critical areas adjacent to the fractured trabecula and endplates, is critical for treatment in these repeat cases. Placement of cement in the areas that are unfilled, especially near the fractured vertebral endplate, can provide significant clinical improvement and definitely will stabilize the progression of these recurrent osteoporotic fractures.

## References

[1] Liu JT, Li CS, Chang CS, Liao WJ (2015). Long-term follow-up study of osteoporotic vertebral compression fracture treated using balloon kyphoplasty and vertebroplasty. J Neurosurg Spine.

[2] Tsai YW, Hsiao FY, Wen YW (2013). Clinical outcomes of vertebroplasty or kyphoplasty for patients with vertebral compression fractures: A nationwide cohort study. J Am Med Dir Assoc.

[3] Frankel BM, Monroe T, Wang C (2007). Percutaneous vertebral augmentation: an elevation in adjacent-level fracture risk in kyphoplasty as compared with vertebroplasty. Spine J.

[4] Lin CC, Shen WC, Lo YC (2010). Recurrent pain after percutaneous vertebroplasty. AJR Am J Roentgenol.

[5] Nieuwenhuijse M, Putter H, van Erkel A (2013). New vertebral fractures after percutaneous vertebroplasty for painful osteoporotic vertebral fractures: a clustered analysis and the relevance of intradiscal cement leakage. Radiology.

[6] Lavelle WF, Chenez R (2006). Recurrent fracture after vertebral kyphoplasty. Spine J.

[7] Wiggins M, Schizadeh M, Pilgram T, Gilula LA (2007). Importance of intravertebral fracture clefts in vertebroplasty outcome. AJR Am J Roentgenol.

[8] Gaughen JR, Jensen ME, Schweickert PA (2002). The therapeutic benefit of repeat percutaneous vertebroplasty at previously treated vertebral levels. AJNR Am J Neuroradiol.

[9] Wu AM, Chi YL, Ni WF (2013). Vertebral compression fracture with intravertebral vacuum cleft sign: pathogenesis, image, and surgical intervention. Asian Spine J.

[10] Eck JC, Nachtigall D, Humphreys SC, Hodges SD (2008). Comparison of vertebroplasty and balloon kyphoplasty for treatment of vertebral compression fractures: a meta-analysis of the literature. Spine J.

[11] Chen LH, Hsich MK, Liao JC (2011). Repeated percutaneous vertebroplasty for refracture of cemented vertebrae. Arch Orthop Trauma Surg.

[12] Weibo YW, Liang D, Yao Z (2017). Risk factors for recollapse of the augmented vertebrae after percutaneous vertebroplasty for osteoporotic vertebral fractures with intravertebral vacuum cleft. Medicine (Baltimore).

[13] Hiwatashi A, Yoshiura K, Yamashita H (2009). Subsequent fracture after percutaneous vertebroplasty can be predicted on preoperative multidetector CT. AJNR Am J Neuroradiol.

[14] Schmidt R, Cakir B, Mattes T (2005). Cement leakage during vertebroplasty: an underestimated problem?. Eur Spine J.

[15] Hatgis J, Granville M, Jacobson RE (2017). Evaluation and interventional management of pain after vertebral augmentation procedures. Cureus.

[16] Pham T, Azulay-Parrado J, Champsaur P (2005). "Occult" osteoporotic vertebral fractures: vertebral body fractures without radiologic collapse. Spine (Phila Pa 1976).

[17] Kim YJ, Chae SU, Kim GD (2013). Radiographic detection of osteoporotic vertebral fracture without collapse. J Bone Metab.

[18] Oh HS, Kim TW, Kim HG, Park KH (2016). Gradual height decrease of augmented vertebrae after vertebroplasty at the thoracolumbar junction. Korean J Neurotrauma.

[19] Zhang L, Wang O, Wang L (2017). Bone cement distribution in the vertebral body affects chances of recompression after percutaneous vertebroplasty treatment in elderly patients with osteoporotic vertebral compression fractures. Clin Interv Aging.

[20] Knavel EM, Rad AE, Thielen KR, Kallmes DF (2009). Clinical outcomes with hemivertebral filling during percutaneous vertebroplasty. AJNR Am J Neuroradiol.

[21] Liu D, Liu XL, Zhang B (2015). Computer simulation and analysis on flow characteristics and distribution patterns of polymethylmethacrylate in lumbar vertebral body and vertebral pedicle. Biomed Res Int.

[22] Erbe EM, Clineff TD, Gualtieri G (2001). Comparison of a new bisphenol-a-glycidyl dimethacrylate-based cortical bone void filler with polymethyl methacrylate. Eur Spine J.

[23] Bae H, Hatten HP Jr, Linovitz R (2012). A prospective randomized FDA-IDE trial comparing Cortoss with PMMA for vertebroplasty: a comparative effectiveness research study with 24-month follow-up. Spine (Phila Pa 1976).

[24] Becker S, Tuschel A, Chavanne A (2008). Balloon kyphoplasty for vertebra plana with and without osteonecrosis. J Orthop Surg (Hong Kong).

[25] Linn J, Birkenmaier C, Hoffmann RT (2009). The intravertebral cleft in acute osteoporotic fractures: fluid in magnetic resonance imaging-vacuum in computerized tomography?. Spine (Phila Pa 1976).

[26] Ishiyama M, Numaguchi Y, Makidono A (2013). Contrast-enhanced MRI for detecting intravertebral cleft formation: relation to the time since onset of vertebral fracture. AJR Am J Roentgenol.

[27] Kim YJ, Lee JW, Kim KJ (2010). Percutaneous vertebroplasty for intravertebral cleft: analysis of therapeutic effects and outcome predictors. Skeletal Radiol.

[28] Granville M, Jacobson RE (2017). An innovative use of Cortoss bone cement to stabilize a nonunion after interbody fusion. Cureus.

[29] Kawaguchi S, Horigome K, Yajima H (2010). Symptomatic relevance of intravertebral cleft in patients with osteoporotic vertebral fracture. J Neurosurg Spine.

[30] Libicher M, Appelt A, Berger I (2007). The intravertebral vacuum phenomen as specific sign of osteonecrosis in vertebral compression fractures: results from a radiological and histological study. Eur Radiol.

[31] Ryu CW, Han H, Lee YM, Lim MK (2009). The intravertebral cleft in benign vertebral compression fracture: the diagnostic performance of non-enhanced MRI and fat-suppressed contrast-enhanced MRI. Br J Radiol.

